# Effect of late sodium current inhibition on MRI measured diastolic dysfunction in aortic stenosis: a pilot study

**DOI:** 10.1186/s13104-016-1874-0

**Published:** 2016-02-04

**Authors:** Anvesha Singh, Christopher D. Steadman, Jamal N. Khan, Giorgio Reggiardo, Gerry P. McCann

**Affiliations:** Department of Cardiovascular Sciences, University of Leicester and NIHR Leicester Cardiovascular Biomedical Research Unit, Glenfield Hospital, Groby Road, Leicester, LE3 9QP UK; Poole Hospital NHS Foundation Trust, Poole, UK; Biostatistics Unit, Medi Service, Genoa, Italy

**Keywords:** Aortic stenosis, Diastolic dysfunction, Ranolazine, Myocardial perfusion reserve

## Abstract

**Background:**

Ranolazine is a new anti-anginal drug that acts via late sodium current inhibition, and has been shown to improve diastolic dysfunction in isolated myocytes. Diastolic dysfuntion is common in patients with aortic stenosis (AS), and precedes symptom development and systolic dysfunction. The purpose of this study was to assess the effects of ranolazine on peak early diastolic strain rate (PEDSR) and exercise capacity in patients with AS.

**Methods:**

Patients with asymptomatic moderate to severe AS and diastolic dysfunction underwent trans-thoracic echocardiography, exercise testing and cardiac magnetic resonance (CMR) imaging at baseline, 6 weeks after commencing ranolazine and at 10 weeks (4 weeks after discontinuation). Diastolic function was assessed using PEDSR measured on tagged CMR images.

**Results:**

Fifteen patients (peak pressure gradient 48.8 ± 12.4 mmHg, mean pressure gradient 27.1 ± 7.5 mmHg, aortic valve area 1.26 ± 0.31 cm^2^) completed the week-6 visit and 13 completed the final visit. Global PEDSR did not significantly increase from baseline (0.79 ± 0.15) to week-6 (0.86 ± 0.18, p = 0.198). There was a borderline significant increase in total exercise duration from 10.47 ± 3.68 min to 11.60 ± 3.25 min (p = 0.06).

**Conclusion:**

This small pilot study did not show a significant improvement in diastolic function with the use of ranolazine in asymptomatic patients with moderate-severe AS. Further studies with a larger population may be indicated.

*EduraCT number 2011*-*000111*-*26*

**Electronic supplementary material:**

The online version of this article (doi:10.1186/s13104-016-1874-0) contains supplementary material, which is available to authorized users.

## Background

Aortic stenosis (AS) is the commonest valve lesion requiring surgery in the developed world [1], and its prevalence is increasing with an ageing population. It is common in the elderly [2], with up to 3 % of the population over 75 years of age having severe AS [3]. The prognosis of symptomatic AS is greatly improved by aortic valve replacement and surgery is universally recommended in this situation [4, 5]. However, many patients remain symptomatic following surgery and there are currently no medical therapies which are of proven value in AS or other conditions characterised by diastolic dysfunction.

The mechanism of symptom generation in AS is unclear. The presence of left ventricular hypertrophy (LVH) and diastolic dysfunction appear to be important determinants of exercise capacity in AS [6–8]. Following surgery, slow improvements in exercise capacity are seen [9], mirroring reductions in LV mass [10–12]. The mechanism of exercise intolerance is probably related to persistent diastolic dysfunction due to incomplete resolution of interstitial myocardial fibrosis [11, 12]. Additionally, subendocardial ischemia occurs in AS patients, even in those with angiographically normal epicardial coronary arteries [13]. Such ischaemia is likely to exacerbate diastolic dysfunction and in turn perpetuate further ischaemia.

Cardiac magnetic resonance imaging (CMR) allows accurate quantification of LV mass, volumes and systolic function. Its also allows measurement of myocardial deformation in the form of strain and strain rates, and peak early diastolic strain rate (PEDSR), which measures the rate of myocardial relaxation, and is a largely load-independent measure of diastolic function [14]. PEDSR has been shown to be a sensitive marker of diastolic dysfunction in patients with type-II diabetes [15], and has been widely used in other CMR studies [16, 17].

Ranolazine is a newly licensed drug for the treatment of chronic stable angina [18, 19]. Ranolazine, through the inhibition of the late sodium (Na^+^) current, decreases intracellular calcium concentration and shortens the action potential duration, without clinically significant effects on heart rate or blood pressure. In experimental models, ranolazine has been shown to improve diastolic dysfunction in isolated myocytes [20–22] and reduce progressive remodelling in a dog model of heart failure [23]. In small pilot studies of patients with angina, ranolazine decreased reversible ischaemia on scintigraphy [24] and improved echocardiographic parameters of diastolic and systolic function [25].

## Objectives

The objectives of this pilot study were to assess the effects of Ranolazine on diastolic dysfunction, myocardial perfusion reserve (MPR) and exercise capacity in asymptomatic patients with moderate to severe AS with evidence of diastolic dysfunction and/or LVH.

## Methods

### Study design

The study was a prospective, single centre, open label, single group, proof-of-concept study, with blinded endpoint analysis (EduraCT number 2011-000111-26). The United Kingdom National Research Ethics Service (Harrow, Reference 11/LO/0553) approved the study and written informed consent was obtained from all subjects before participation. All imaging was analysed blinded to patient or visit information and the anonymised data was sent to the clinical trials unit (CTU), for unblinding and analysis at the end of the study.

### Study population

Patients were prospectively recruited from a single Cardiac centre. Inclusion criteria were: (1) moderate or severe AS (two or more of aortic valve area <1.5cm2, peak pressure gradient >36 mmHg, mean pressure gradient >20 mmHg), (2) asymptomatic, (3) evidence of diastolic dysfunction (MV inflow: E/A <1 or TDI: septal E/e’ >15, lateral E/e’ >10 on echocardiogram) or LVH (maximum wall thickness >13 mm on echocardiography), (4) age >18 years. Exclusion criteria were: (1) history of coronary artery bypass graft, myocardial infarction or angiographic coronary artery disease (>50 % luminal stenosis if previously undertaken), (2) atrial fibrillation, (3) severe asthma (4) severe renal impairment (eGFR <30 ml/min), (5) hepatic impairment, (6) Concurrent administration of strong CYPA4 inhibitors or class I/III anti-arrhythmic agents, (7) QTc prolongation >470 ms, (8) Females of childbearing potential, (9) Use of >20 mg of simvastatin or >1000 mg of metformin during the study period.

### Study conduct

**Fig. 1:**
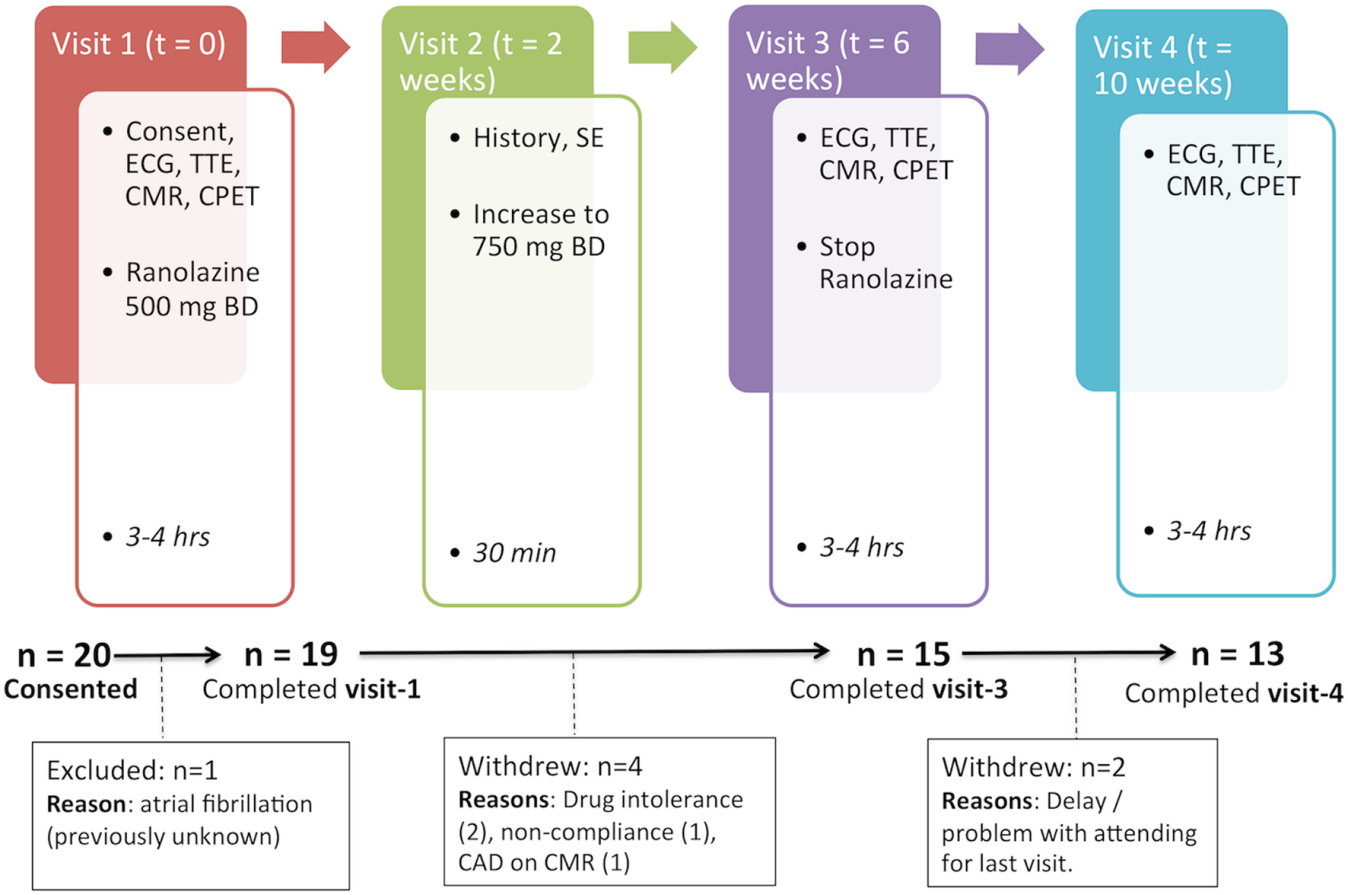
Study overview and recruitment (*ECG* electrocardiogram, *TTE* trans-thoracic echocardiogram, *CMR* cardiac magnetic resonance imaging, *ET* exercise test, *SE* side effects, *CAD* coronary artery disease)

Consented patients underwent venepuncture, electrocardiography (ECG), trans-thoracic echocardiography, exercise testing and stress CMR, before being commenced on 500 mg bd of ranolazine (C_24_H_33_N_3_O_4_, Gilead Sciences). This was up-titrated to 750 mg bd after 2 weeks, if tolerated, and continued for another 4 weeks. The study involved four outpatient visits to the hospital as outlined in Fig. 1. All investigations were repeated at week-6 and again at week-10 (4 weeks after stopping ranolazine).

### Echocardiography

Echocardiography was performed according to the American Society of Echocardiography recommendations [26], and blinded, off-line analysis was performed using Xcelera v3.3.1 (Philips, Netherlands) workstation. In addition to the standard 2D, colour flow and Doppler images to assess AS severity and LV function, diastolic function was assessed using pulsed-wave Doppler and tissue Doppler, to get the E-wave, A-wave, E/A and septal and lateral E/e’. Continuous-wave Doppler data was acquired at rest as well as post-exercise. The left ventricular rate pressure product (LVRPP), a surrogate marker of myocardial oxygen consumption, was calculated at rest and at peak exercise using the following formula:

$$LVRPP\,=\,(PPG\,+\,SBP)\,\times\,HR$$

(*LVRPP* left ventricular rate pressure product in mmHg.bpm, *PPG* peak aortic valve pressure gradient in mmHg, *SBP* peak systolic blood pressure in mmHg; *HR* heart rate in beats per minute).

### Exercise testing

A treadmill test was performed, with continuous ECG monitoring, using the modified Bruce Protocol. The subjects were exercised till they had achieved at least 85 % of their maximal predicted heart rate, a limiting symptom (chest pain, significant dyspnoea or dizziness) or another pre-specified reason for stopping (ST depression >5 mm, significant arrhythmia, systolic blood pressure (SBP) >250 mmHg or diastolic blood pressure >120 mmHg or a fall in SBP >20 mmHg).

### CMR acquisition

**Fig. 2:**
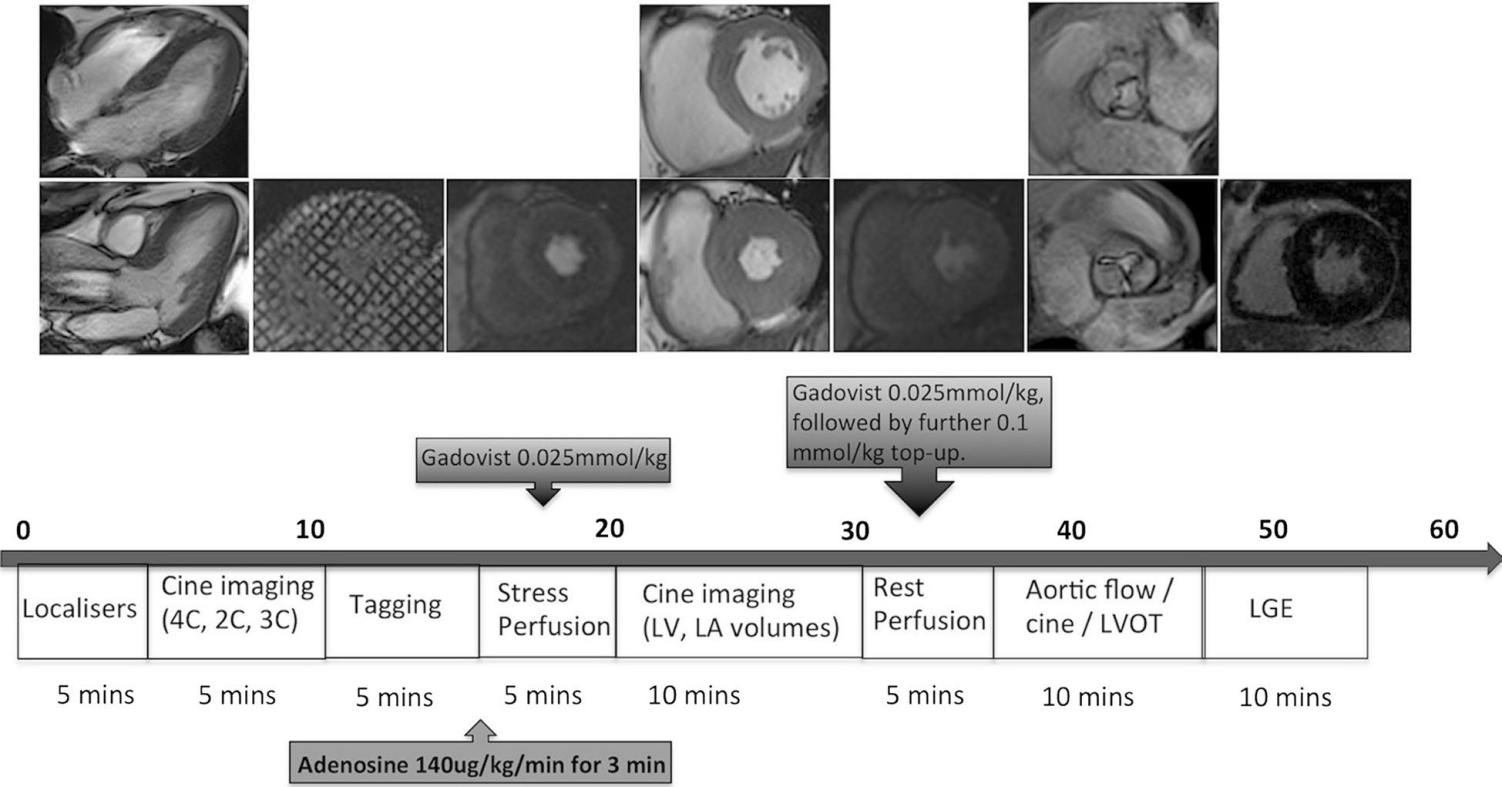
Cardiac MRI protocol used (*4/2/3 C* 4/2/3 chamber, *LV* left ventricular, *LA* left atrial, *LVOT* left ventricular outflow track, *LGE* late gadolinium enhancement)

CMR was performed on a 3-tesla (T) scanner (Magnetom Skyra, Siemens AG, Healthcare Sector, Erlangen, Germany) using an 18-channel phased array receiver coil (Fig. 2). Steady state free precession end-expiratory breath-held cine images were acquired, with retrospective ECG triggering, to determine LV volumes, mass and function. Tagged images were acquired at three short-axis slices (basal, mid, apical), using spatial modulation of magnetization (SPAMM): slice thickness 8 mm, grid tag spacing 8 mm, TR 3.6 ms, TE 2.4 ms, flip angle 10°, temporal resolution 46 ms and prospective gating as previously described [27]. Stress imaging was performed at the same three short-axis slice positions after inducing pharmacological vasodilation with an infusion of adenosine at 140 mg/kg/min for 3 min or until a haemodynamic response and/or symptoms were achieved. First pass perfusion imaging was performed with 0.025 mmol/kg of contrast (Gadovist, Bayer Pharma AG, Germany) at stress and again after 10 min of rest, using a saturation recovery gradient-echo sequence, during breath holding. This was followed by a top-up of 0.1 mmol/kg to bring the total dose of contrast to 0.15 mmol/kg, before late gadolinium imaging (LGE) was performed after a delay of 10 min.

### CMR analysis

CMR42 v4.2 (Circle Cardiovascular Imaging, Calgary, Alberta, Canada) was used to calculate the LV mass and volume data. Circumferential peak systolic strain (PSS), peak systolic strain rate (PSSR) and peak early diastolic strain rate (PEDSR) were calculated for each slice and globally (average of base, mid, apex) using the InTag post-processing plugin (Creatis, Lyon, France) for OsiriX (Geneva, Switzerland) on the tagged images as previously described [27]. The segmental strain outputs generated by the software were post-processed using in-house Microsoft excel spreadsheets (Microsoft Office 2011, California, USA), in order to obtain average strain and strain rate curves for each slice. Perfusion analysis was performed using QMass v7.1 (Medis Medical Imaging Systems, Netherlands), to produce graphs of signal intensity against time. Absolute myocardial blood flow (MBF) quantification was performed as previously described [28, 29], using model-independent deconvolution. Myocardial perfusion reserve (MPR) was calculated by dividing hyperaemic MBF by resting MBF.

### Endpoints

The primary hypothesis was that late sodium current inhibition with ranolazine would improve PEDSR, a measure of diastolic dysfunction, on tagged MRI. Secondary endpoints included MPR, exercise capacity and echocardiographic markers of diastolic function (E/E’).

### Statistical analysis and power calculation

In a previous group of eight AS patients tested 2 weeks apart, diastolic strain rate was 0.73 ± 0.22 and 0.71 ± 0.21 with paired mean difference of 0.04 and SD of 0.16. Sixteen patients with analysable images would allow us to detect a difference of 0.12 in diastolic strain rate with 80 % power, p < 0.05 and two-tailed. To allow for drop-outs and unanalysable image quality, we planned to recruit 20 patients. Statistical tests were performed using SPSS 20.0 software (Statistical Package for the Social Sciences, Chicago, IL). Normality was assessed using the Shapiro–Wilk test, histograms and Q–Q plots. Continuous data are expressed as mean (standard deviation). Paired-samples *t* tests were used to compare parameters between different visits. In addition, repeated measure analysis of variance (ANOVA) was used to compare parameters across the three visits. The results were further analysed after splitting the patients according to the median MPR, into low and high-MPR subgroups, and patient characteristics between the two sub-groups were compared using independent *t* test. Two-factor repeated measure ANOVA analysis was used to compare change in global PEDSR between the two sub-groups.

## Results

Twenty patients consented to take part in the study but one patient was excluded after ECG showed atrial fibrillation. Nineteen completed the baseline visit, 4 withdrew before visit-3 and therefore, a total of 15 patients had data for the primary endpoint analysis at 6 weeks (Fig. 1). The demographic data for these patients (as well as the sub-groups) is shown in Table 1. Two continued on the lower dose of ranolazine (500 mg BD) due side effects with the higher dose. Another two patients were unable to attend the week-10 visit, making the full analysis sample (FAS) consist of 13 patients.

**Table 1:** Demographic data for overall study population and high and low-MPR subgroups

Parameter	Overall (n = 15)	Low-MPR (n = 7)	High-MPR (n = 8)
Age (years)	65.9 ± 9.67	63.6 ± 10.3	67.9 ± 9.3
Gender ratio (male/female, n (%))	12/3 (80.0/20.0)	6/1 (85.7/14.3)	6/2 (75.0/25.0)
BMI (kg/m^2^)	29.3 ± 3.36	29.3 ± 4.3	29.3 ± 2.6
Heart rate (bpm)	74.5 ± 11.8	78.6 ± 9.4	71.0 ± 13.2
Systolic blood pressure (mmHg)	153.0 ± 23.6	153.0 ± 29.3	153.0 ± 19.6
Diastolic blood pressure (mmHg)	81.7 ± 11.1	83.7 ± 11.3	80.0 ± 11.4
Echocardiographic Data			
Peak pressure gradient (mmHg)	48.8 ± 12.4	50.5 ± 13.0	49.2 ± 12.7
Mean pressure gradient (mmHg)	27.1 ± 7.5	27.4 ± 7.5	27.0 ± 8.1
Aortic valve area (cm^2^)	1.26 ± 0.31	1.31 ± 0.40	1.20 ± 0.21
E/A	0.77 ± 0.16	0.79 ± 0.17	0.76 ±0.17
Average septal E/e’	12.94 ± 3.91	11.19 ± 1.01	14.48 ± 4.89
Average lateral E/e’	10.65 ± 3.49	9.34 ± 2.90	11.80 ± 3.73
Resting LVRPP (mmHg.bpm)	14,424.3 ± 3054.0	15,400.5 ± 2627.3	13,570.2 ± 3309.1
Exercise LVRPP (mmHg.bpm)	36,041.3 ± 5235.1	38,888.7 ± 4365.0	34,449.0 ± 5656.8
CMR data			
LVMI (g/m^2^)	66.72 ± 15.35	68.2 ± 13.9	65.4 ± 17.3
LVEDVI (ml/m^2^)	85.02 ± 15.92	81.59 ± 17.33	88.03 ± 15.07
LVEF (%)	58.29 ± 3.81	58.3 ± 3.3	58.3 ± 4.4

*BMI* body mass index, *LVRPP* left ventricular rate pressure product, *LVMI* left ventricular mass indexed to body surface area, *LVEDVI* left ventricular end diastolic volume indexed to body surface area, *LVEF* left ventricular ejection fraction

Independent *t* test used to compare low and high-MPR subgroups

### Primary endpoint: PEDSR

In these data, there is insufficient evidence that global PEDSR changed significantly from the baseline value to week-6, although the mean PEDSR increased numerically (Table 2). A similar pattern was demonstrated for each slice individually (change in PEDSR from baseline to week-6 from 0.818 to 0.893 for basal, 0.829 to 0.841 for mid and 0.756 to 0.790 for apical slices). For the FAS sample, global PEDSR was: baseline (0.82 ± 0.13), week-6 (0.87 ± 0.19), and week-10 (0.81 ± 0.21) (p > 0.05 using both paired *t* tests and repeated measures ANOVA analysis) (Additional file 1: Table S1).

**Table 2:** Primary and secondary endpoint measures: baseline *vs* week-6 (n = 15)

Parameter	Baseline	Week-6	p (paired t test)
MRI parameters			
PEDSR (1/s)	0.79 ± 0.151	0.86 ± 0.181	0.198
PSS (%)	−17.44 ± 2.57	−17.53 ± 3.98	0.907
PSSR (1/s)	−0.99 ± 0.203	−1.04 ± 0.208	0.436
MPR	2.68 ± 0.634	2.52 ± 0.614	0.452
LVEDV (ml)	173.7 ± 47.64	170.1 ± 59.02	0.624
LVESV (ml)	73.3 ± 25.26	74.3 ± 29.38	0.509
EF (%)	58.3 ± 3.81	56.7 ± 4.81	0.080
Exercise parameters			
Resting HR (bpm)	74.5 ± 11.8	74.4 ± 13.7	0.963
Resting SBP (mmHg)	153.0 ± 23.6	147.2 ± 17.3	0.208
Exercise duration (min)	10.47 ± 3.68	11.60 ± 3.25	0.062
Max HR (bpm)	143.5 ± 10.7	139.6 ± 15.5	0.273
Max SBP (mmHg)	182.9 ± 20.5	174.5 ± 25.8	0.133
Resting LVRPP (mmHg.bpm)	14,424.3 ± 3054.0	14,514.1 ± 3591.6	0.903
Exercise LVRPP (mmHg.bpm)	36,041.3 ± 5235.1	34,516.9 ± 6538.4	0.313
Biomarker			
NT-proBNP (pmol/L)	48.54 ± 82.43	51.64 ± 73.28	0.715
Echocardiographic parameters			
E/A	0.773 ± 0.163	0.783 ± 0.169	0.765
Septal E/e’	12.94 ± 3.91	13.79 ± 2.86	0.258
Lateral E/e’	10.65 ± 3.49	10.62 ± 3.43	0.979

Abbreviations as Table 1. *PEDSR* peak early diastolic strain rate, *PSS* peak systolic strain, *PSSR* peak systolic strain rate, *MBF* myocardial blood flow, *MPR* myocardial perfusion reserve, *LVESV* left ventricular end systolic volume, *HR* heart rate, *SBP* systolic blood pressure. All p > 0.05

### Secondary endpoints

**Fig. 3:**
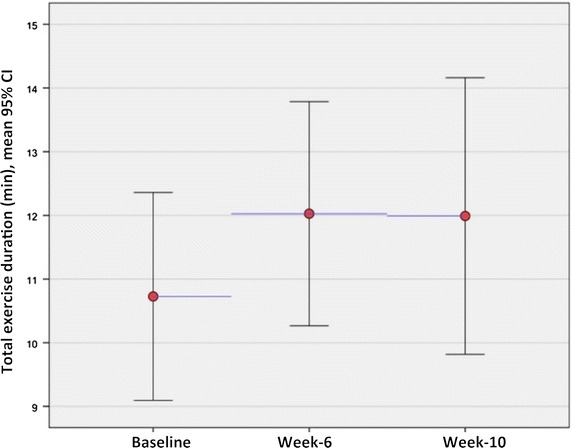
Total exercise duration for all patients exercised at each visit (p = 0.07 for baseline *vs* week-6, p = 0.73 for week-6 vs week-10)

The results of the secondary endpoints are shown in Table 2. There was no significant change in global MPR or echocardiographic measures of diastolic dysfunction. The total exercise duration increased from 10.47 ± 3.68 min to 11.60 ± 3.25 min at week-6 (n = 15), which was of borderline significance (p = 0.06), but remained elevated at week-10 (Additional file 2: Table S2). Figure 3 shows the total exercise duration for all patients at each visit (baseline: n = 19, week-6: n = 15, week-10: n = 13). The maximal HR and SBP tended to be lower at week-6, leading to a non-significant reduction in exercise LVRPP at week-6. There was no change in the resting values of HR, SBP or LVRPP.

### Subgroup analysis by MPR

**Fig. 4:**
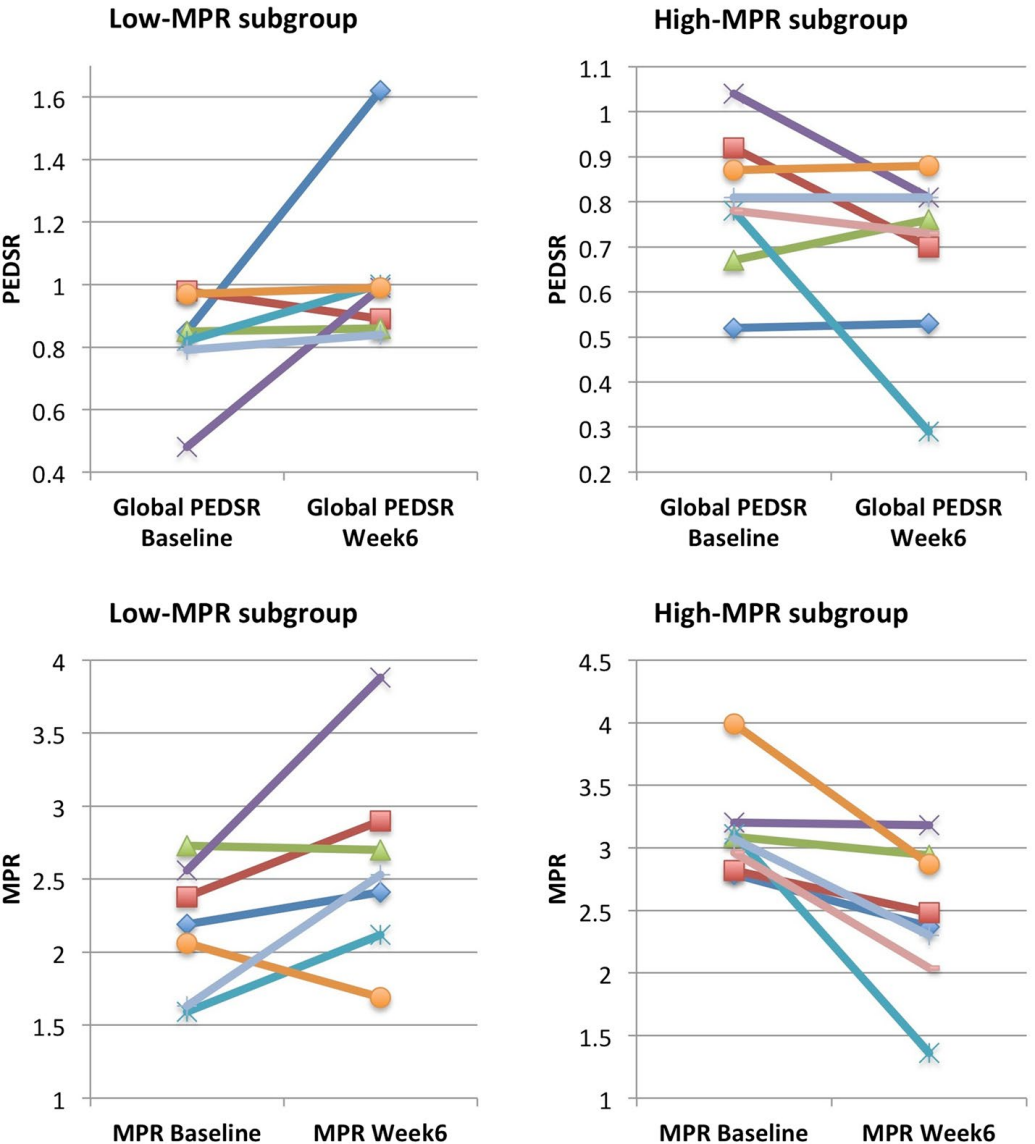
Change in peak early diastolic strain rate (*PEDSR*-*top panels*) and myocardial perfusion reserve (*MPR*-*bottom panels*) in low-MPR (*left*) and high-MPR (*right*) subgroup

The patients were divided into low and high-MPR subgroups based on the median MPR of 2.79. The groups were well matched for age, gender, AS severity and resting haemodynamic data (Table 1). There was a non-significant increase in global PEDSR from baseline to week-6 in the low-MPR sub-group (0.82 ± 0.17 to 1.03 ± 0.27, p = 0.139), which was not present in the high-MPR subgroup (Fig. 4), though this difference remained non-significant (p > 0.05) on two-factor repeated measures ANOVA analysis. Table 3 shows the results of the PEDSR and exercise data for the FAS sample, according to the sub-groups, which were not statistically significant.

**Table 3:** Sub-group analysis of FAS according to MPR

Parameter	Baseline	Week-6	Week-10
Group A (Low-MPR)			
Global PEDSR	0.88 ± 0.80	1.03 ± 0.30	0.86 ± 0.15
Exercise time (min)	11.0 ± 3.6	11.2 ± 3.8	11.5 ± 3.6
Exercise LVRPP (mmHg.bpm)	39,148.9 ± 3052.2	35,417.1 ± 7042.9	36,882.7 ± 5799.2
Group B (High-MPR)			
Global PEDSR	0.80 ± 0.19	0.75 ± 0.12	0.76 ± 0.28
Exercise time (min)	11.8 ± 3.7	11.7 ± 3.7	11.8 ± 4.2
Exercise LVRPP (mmHg.bpm)	33,615.0 ± 6402.4	33,085.4 ± 7839.0	34,357.3 ± 4188.5

Abbreviations as Table 1. All p > 0.05. Low MPR = MPR < 2.79, n = 7; High MPR = MPR ≥ 2.79, n = 8)

## Discussion

### PEDSR

This single-centre pilot study aimed to assess the effects of ranolazine on diastolic function in patients with moderate to severe AS and evidence of diastolic dysfunction. There was no evidence in these data that ranolazine improved PEDSR.

The effect of ranolazine in improving diastolic function has previously been demonstrated in both animal models [21, 30] and experimental in vitro studies in human myocytes [20, 22]. The exact mechanism for this effect is not entirely clear, but is thought to be related to the late Na^+^ current inhibition by ranolazine, leading to a decrease in intra-cellular-Na^+^ dependent intracellular calcium concentration [20]. There have only been a few small clinical studies assessing the effect of ranolazine on diastolic function. In a study of ischaemic heart disease patients with previous MI (n = 15), Ranolazine infusion improved regional diastolic function, measured by 2-dimensional invasive LV angiograohy in non-infarcted ischaemic segments [31]. Another small study of patients with stable angina (n = 22) demonstrated improvement in some echocardiographic parameters of diastolic function, but not others [25]. A case report documented improvement in ischaemic burden and symptoms in a patient with patent grafts but on-going ischaemia, most likely due to microvascular and diastolic dysfunction [32]. Finally, a recently published randomised trial of ranolazine in patients with heart failure with preserved EF (n = 20) failed to demonstrate improvement in echocardiographic measures of diastolic function [33]. A limitation in all the above-mentioned studies may be their small sample sizes.

The lack of statistical significance in our study may not reflect a lack of efficacy. The sample size of the study was small, with only fifteen patients having analysable tagging images for the primary endpoint, compared to an anticipated 16 at inception. Since the study commenced, we have also shown that PEDSR may be less reproducible using tagging (SPAMM) at 3T, as used in this study, compared to CSPAMM tagging at 1.5T that was used to estimate the sample size [27]. Additionally, on discontinuation, mean PEDSR tended to return towards the baseline value, suggesting a possible genuine effect of ranolazine. Sub-group analysis also demonstrated interesting differences, with mean PEDSR showing some improvement in the low-MPR subgroup- albeit this effect was only striking in two patients, and may represent chance (Fig. 4). The suggestion that ranolazine may have greater efficacy in improving diastolic function in those with more advanced disease (reduced perfusion reserve [28]) and ischaemia is purely a hypothesis at this stage. Finally, the maximum dose of ranolazine used in our study was 750 mg bd (with two patients continuing on 500 mg bd), which is lower than the dose of 1000 mg bd used in most other studies mentioned above, which may partially account for the lack of efficacy.

### MPR

Our study did not demonstrate improvement in global MPR in the overall population following 6 weeks of ranolazine therapy. In a previous open-label pilot study of patients with coronary artery disease and perfusion defects on exercise SPECT myocardial perfusion imaging (n = 20), 4 weeks of treatment with ranolazine led to an improvement in myocardial perfusion pattern and severity [24]. This is thought to occur due to reduced diastolic wall stiffness caused by the late Na^+^ current inhibition by ranolazine, leading to reduced extra-vascular compression of the coronary microcirculation, and improved myocardial blood flow. However, another study by the same group using vasodilator stress (n = 18), failed to show an improvement in myocardial perfusion [34]. Exercise testing induces true ischaemia by a supply/demand mismatch, whereas vasodilator-stress induced perfusion defects result from regional heterogeneity in blood flow, which may not activate the late Na^+^ current. In a more recent pilot study of patients with microvascular angina, ranolazine did not lead to an improvement in coronary flow reserve, measured by Doppler echocardiography, in response to adenosine or cold pressor test [35].

### Exercise capacity

The total exercise time did show an increase at week-6, which was close to reaching statistical significance. This observation lends weight to the hypothesis that the small increase in PEDSR seen may be significant. We cannot discount that the increase in exercise capacity was related to improved technique on the treadmill, as there was no reduction in duration at week-10. However, the increase in exercise duration at week-6 was associated with a slightly reduced peak heart and blood pressure compared to baseline, and therefore a slightly lower exercise LVRPP (a measure of myocardial work), that were not sustained at week 10.

Ranolazine may increase myocardial efficiency during exercise, and the mechanism for this may be related to an improvement in PEDSR. Ranolazine has previously been shown to increase exercise duration in multiple studies of patients with chronic stable angina [18, 19, 36]. In the study of patients with HFpEF, ranolazine improved the VE/VCO2 slope, an index of ventilatory response to exercise, as well as the exercise duration [33].

### Limitations

The major limitation of this study is its small sample size, with the results not reaching statistical significance. However the study was always planned as a pilot and the primary purpose was to assess the effect size. This hypothesis-generating study has certainly shown some interesting signals that merit further investigation in larger trials.

## Conclusions

In this pilot study, ranolazine did not show a significant improvement in diastolic dysfunction or MPR, in patients with moderate to severe AS. Given the low power of the current study, a larger study in patients with diastolic dysfunction is warranted and preferably conducted at 1.5T.

## Availability of supporting data

The dataset supporting the results of this article is included in this article and its supporting file.

## Additional files

10.1186/s13104-016-1874-0 Tagging measured PEDSR for FAS population: baseline *vs*. week-6 *vs*. week-10 (n=13).
10.1186/s13104-016-1874-0 Secondary endpoint measures for FAS population: baseline *vs*. week-6 *vs*. week-10 (n=13).

## Abbreviations

AS: aortic stenosis
CTU: clinical trials unit
ECG: electrocardiography
FAS: full analysis set
HR: heart rate
LGE: late gadolinium enhancement
LV: left ventricle
LVH: left ventricular hypertrophy
LVRPP: left ventricular rate pressure product
MBF: myocardial blood flow
MPR: myocardial perfusion reserve
MRI: magnetic resonance imaging
Na^+^: sodium
PEDSR: peak early diastolic strain rate
PSS: peak systolic strain
PSSR: peak systolic strain rate
SBP: systolic blood pressure
