# Supplementary material for: Effect of late sodium current inhibition on MRI measured diastolic dysfunction in aortic stenosis: a pilot study
Source: BMC Res Notes. 2016 Feb 4;9:64. doi: 10.1186/s13104-016-1874-0 (PMC4743087; doi:10.1186/s13104-016-1874-0)
Supplement: Supplementary file 1 — 10.1186/s13104-016-1874-0 Tagging measured PEDSR for FAS population: baseline vs. week-6 vs. week-10 (n=13). [file 13104_2016_1874_MOESM1_ESM.docx]

Table S1. Tagging measured PEDSR for FAS : Baseline *vs* Week-6 *vs* Week-10 (n=13)

| **Slice** | **Baseline** | **Week-6** | **Week-10** |
| --- | --- | --- | --- |
| **Base** | 0.860 ± 0.206 | 0.939 ± 0.281 | 0.899 ± 0.282 |
| **Mid** | 0.839 ± 0.140 | 0.851 ± 0.155 | 0.760 ± 0.184 |
| **Apex** | 0.793 ± 0.229 | 0.795 ± 0.251 | 0.751 ± 0.261 |
| **Global** | 0.82 ± 0.130 | 0.87 ± 0.193 | 0.81 ± 0.211 |

^All p-values >0.05^

Table S2. Secondary endpoint measures FAS baseline *vs* Week-6 *vs* Week-10 (n=13)

| **Parameter** | **Baseline** | **Week-6** | **Week-10** |  |
| --- | --- | --- | --- | --- |
| **MRI Parameters** | | | |  |
| **PSS** (%) | -18.05 ± 2.43 | -18.93 ± 2.63 | -18.57 ± 3.38 |  |
| **PSSR** (1/s) | -1.01 ± 0.21 | -1.08 ± 0.21 | -1.00 ± 0.18 |  |
| **MPR** | 2.69 ± 0.726 | 2.45 ± 0.559 | 2.52 ± 0.579 |  |
| **LVEDV** (ml) | 174.31 ± 44.17 | 173.08 ± 60.22 | 172.00 ± 59.58 |  |
| **LVESV** (ml) | 72.46 ± 22.47 | 74.69 ± 29.89 | 74.15 ± 26.14 |  |
| **Exercise Parameters** | | | |  |
| **Resting HR** (bpm) | 74.1 ± 12.0 | 73.5 ± 13.2 | 72.0 ± 11.7 |  |
| **Resting SBP** (mmHg) | 155.9 ± 24.0 | 148.5 ± 17.9 | 146.2 ±25.0 |  |
| **Exercise duration** (min) | 10.88 ± 3.94 | 11.85 ± 3.39 | 11.99 ± 3.59 |  |
| **Max HR** (bpm) | 142.2 ± 11.0 | 136.5 ± 13.7 | 140.6 ± 12.7 |  |
| **Max SBP** (mmHg) | 183.9 ± 20.6 | 174.5 ± 24.2 | 179.3 ± 15.1 |  |
| **Resting LVRPP** (mmHg.bpm) | 14639.7 ± 3228.3 | 14735.9 ± 3773.6 | 14512.6 ± 3131.8 |  |
| **Exercise LVRPP** (mmHg.bpm) | 36342.3 ± 5351.4 | 34285.9 ± 6902.7 | 35604.6 ± 4787.5 |  |
| **Echocardiographic Parameters** | | | |  |
| **E/A** | 0.742 ± 0.143 | 0.765 ± 0.171 | 0.775 ± 0.159 |  |
| **Septal E/e’** | 12.57 ± 3.81 | 13.50 ± 2.95 | 13.84 ± 3.85 |  |
| **Lateral E/e’** | 10.93 ± 3.48 | 10.90 ± 3.37 | 11.03 ± 2.75 |  |

^Abbreviations as Table-2.^
